# Factors Associated with Transplant-Specific Psychological Adaptation Among Heart and Kidney Transplant Recipients

**DOI:** 10.3390/healthcare14203551

**Published:** 2026-10-21

**Authors:** Irena Milaniak, Roksana Pawlikowska, Grażyna Dębska

**Affiliations:** 1 Faculty of Health Sciences, Andrzej Frycz Modrzewski Krakow University, 30-705 Kraków, Poland; 74220@student-afm.edu.pl; 2 Medical Institute, University of Applied Sciences in Nowy Targ, 34-400 Nowy Targ, Poland; grazyna.debska@ans-nt.edu.pl

**Keywords:** transplantation, psychosocial assessment, adaptation

## Abstract

**Highlights:**

**What are the main findings?**

• Several psychological variables were associated with transplant-specific psychological adaptation in heart and kidney transplant recipients.
• Better mental health-related quality of life was independently associated with greater treatment adherence among heart transplant recipients.

**What are the implications of the main findings?**

• Routine assessment of transplant-specific psychological adaptation may help identify recipients requiring additional psychosocial support.
• Routine psychosocial assessment may help identify recipients requiring additional psychosocial evaluation and should be investigated in future intervention studies.

**Abstract:**

Background: Psychological adaptation after solid organ transplantation extends beyond graft survival and includes transplant-specific emotional responses that may influence long-term health outcomes. However, factors associated with transplant-specific psychological adaptation remain insufficiently understood, particularly in Central European populations. Methods: A multicentre cross-sectional study was conducted among 224 adult heart (*n* = 107) and kidney (n = 117) transplant recipients attending three Polish transplant centers. Participants completed the Transplant Effects Questionnaire (TxEQ), Hospital Anxiety and Depression Scale (HADS), General Self-Efficacy Scale (GSES), Life Orientation Test-Revised (LOT-R), and SF-36. We performed univariable and multivariable linear regression analyses separately for heart and kidney transplant recipients to identify factors associated with transplant-specific psychological adaptation. Results: Overall psychological adaptation was favorable. Adherence demonstrated the highest TxEQ scores, whereas donor-related guilt and disclosure concerns were relatively low. Approximately 20.53% of participants reported clinically relevant anxiety symptoms and 14.2% reported depressive symptoms. In heart transplant recipients, better mental health-related quality of life was independently associated with greater adherence (β = 0.025, *p* = 0.003). Among kidney transplant recipients, higher donor-related guilt was associated with transplantation center, while greater disclosure was associated with male sex, transplantation center, and shorter time since transplantation (all *p* < 0.05). Several psychological variables were associated with selected domains of transplant-specific psychological adaptation, whereas demographic and clinical variables showed fewer significant associations. Conclusions: These findings indicate that several psychological variables were associated with transplant-specific psychological adaptation in long-term heart and kidney transplant recipients. Routine assessment of anxiety, mental health-related quality of life, and transplant-specific emotional responses may facilitate identification of recipients who could benefit from further psychosocial evaluation. Prospective studies are needed to determine whether these associations translate into improved long-term outcomes.

## 1. Introduction

### 1.1. Clinical Background and the Evolution of Transplantation Outcomes

Solid organ transplantation has become the standard treatment for selected patients with end-stage organ failure, offering substantial improvements in survival, functional status, and life expectancy. Advances in surgical techniques, donor management, organ preservation, immunosuppressive therapy, and multidisciplinary follow-up have transformed transplantation from a life-saving intervention into a chronic disease management strategy. Consequently, the growing population of transplant recipients has shifted clinical attention beyond traditional outcomes such as graft survival and mortality towards broader patient-centered outcomes that better reflect long-term recovery and successful reintegration into everyday life. Health-related quality of life, psychosocial functioning, treatment adherence, and patient-reported outcome measures (PROMs) are now recognized as essential indicators of transplantation success and are increasingly incorporated into clinical practice and research [1,2,3,4].

This evolution reflects a fundamental shift in how we understand post-transplant care. Although transplantation effectively restores organ function and alleviates many symptoms associated with end-stage disease, recipients continue to face lifelong medical and psychosocial challenges. Long-term immunosuppressive therapy, the risk of acute and chronic graft rejection, treatment-related adverse effects, repeated medical monitoring, and uncertainty regarding future health require continuous adaptation rather than a simple return to pre-illness functioning. Contemporary models of transplant care therefore emphasize that successful transplantation is a dynamic process in which physical recovery and psychosocial adaptation develop simultaneously. Accordingly, long-term follow-up should address not only biomedical stability but also recipients’ emotional well-being, self-management abilities, social participation, and perceived quality of life.

Growing evidence indicates that psychological adaptation after transplantation is considerably more complex than the absence of anxiety or depression. Recipients frequently experience transplant-specific emotional responses that evolve throughout the post-transplant trajectory and may persist for many years despite satisfactory graft function. Recent longitudinal studies consistently demonstrate that although symptom burden decreases during follow-up, fatigue remains one of the most persistent barriers to recovery [5,6]. Similarly, Almgren et al. reported that persistent fatigue was closely associated with reduced self-efficacy, whereas Lindberg et al. described adaptation as a gradual transition from uncertainty to acceptance [7,8]. Similarly, uncertainty regarding graft survival, fear of rejection, concerns about long-term health, altered body perception, and the need to integrate the transplanted organ into one’s personal identity continue to influence psychological adjustment long after surgery [7,8,9]. Recent qualitative and longitudinal investigations further suggest that adaptation after transplantation is not a single event but an ongoing developmental process. Recipients gradually move from the uncertainty and vulnerability of the early post-transplant period toward greater acceptance, confidence, and social reintegration. However, this trajectory varies widely and is shaped by demographic, clinical, psychological, and social factors. Fatigue is a significant barrier to self-efficacy and participation in daily activities, while persistent symptom burden may compromise emotional well-being despite otherwise favorable clinical outcomes [6,7,10]. Likewise, social adaptation and the reconstruction of personal identity have emerged as fundamental dimensions of long-term recovery, highlighting that transplantation is not only a medical intervention but also a profound biographical transition requiring ongoing psychological adjustment [8,11]. While many psychological issues are common among solid-organ recipients, heart transplant patients experience particularly high symptom burdens. They face an increased risk of developing new mood and anxiety disorders within the first year. Long-term studies indicate that distress related to body image changes, magical thinking about inheriting the donor’s personality, fear of death, as well as financial and family difficulties, are frequent concerns. Constantly focusing on the risk of allograft rejection and the immunosuppressive medication regimen can contribute to anxiety and depression. Additionally, the incidence of post-traumatic stress disorder is notably higher [12].

Research on kidney transplantation shows that recipients often feel liberation and gratitude but also face psychological challenges like depression, anxiety, and fear. The process of psychological adjustment after a transplant is complex, affected by acceptance of the new organ, medication side effects, and ongoing worries about organ rejection [13].

These findings have important implications for clinical practice. As survival after heart and kidney transplantation continues to improve, identifying recipients experiencing difficulties in psychosocial adaptation has become an essential component of comprehensive transplant care. Early recognition of emotional distress, impaired self-management, maladaptive coping strategies, and reduced quality of life may facilitate timely psychosocial interventions, optimize adherence to immunosuppressive treatment, and ultimately improve long-term patient outcomes. Within multidisciplinary transplant programs, transplant nurses are uniquely positioned to monitor these domains during routine follow-up, reinforcing the need for reliable and clinically meaningful assessment of transplant-specific psychological functioning.

### 1.2. Psychological Adaptation, TxEQ, Current Evidence and Knowledge Gap

Beyond general psychosocial adjustment, transplant recipients experience a range of transplant-specific emotional responses that are not adequately captured by conventional psychological measures. Existing literature indicates that transplant recipients often face numerous stressors and psychological challenges associated with the transplantation process. Psychological functioning includes both general psychological well-being (e.g., anxiety, depression, optimism, and self-efficacy) and transplant-specific emotional responses, including worry, guilt, disclosure, responsibility, and adherence. These may include adverse effects of lifelong immunosuppressive therapy, fear of graft rejection, uncertainty regarding the functioning of the transplanted organ, and the need to comply with complex and demanding medical regimens. In addition, psychosocial issues such as changes in family relationships, physical and psychological adjustment after transplantation, integration of a modified body image and feelings of gratitude or guilt toward the donor or donor’s family have been widely documented [4,14]. In this context, previous studies have highlighted psychological integration of the transplanted organ as a key element of successful post-transplant adaptation [3,14]. Due to the persistent stress and uncertainty associated with transplantation, emotional difficulties such as anxiety, depression, and sleep disturbances are frequently reported both before and after transplantation [4,15]. Recent evidence consistently indicates that transplant recipients experience both general psychological distress and transplant-specific emotional responses. While anxiety and depressive symptoms are among the most frequently reported outcomes, several authors have demonstrated that transplant-specific experiences—including donor-related guilt, responsibility for the graft, and disclosure concerns—represent distinct psychological constructs requiring dedicated assessment [16,17]. One instrument designed specifically to capture transplant-related psychological responses is the Transplant Effects Questionnaire (TxEQ). The TxEQ assesses key psychological reactions to receiving a transplanted organ, including worry about the transplant, guilt toward the donor, disclosure of transplantation, adherence to medical treatment, and perceived responsibility toward the donor, family, and transplant team [18]. Accumulating evidence suggests that transplant recipients’ psychological adjustment is influenced not only by medical factors but also by individual psychological resources. Based on previous evidence, optimism, self-efficacy, and health-related quality of life were selected because they represent modifiable personal resources consistently associated with adaptation to chronic illness and post-transplant self-management. Variables such as quality of life, symptoms of anxiety and depression, and dispositional optimism appear to play an important role in adaptation to life after transplantation [19,20,21]. Dispositional optimism, defined as a generalized expectation that positive outcomes will occur, has been associated with better psychological adjustment, more adaptive coping strategies, and improved health outcomes in individuals with chronic illnesses [22,23]. However, the relationship between transplant-specific psychological responses measured by the TxEQ and dispositional optimism remains insufficiently explored. Previous research has instead focused primarily on other personal resources, such as sense of coherence, which have been linked to better psychological functioning among transplant recipients [24]. Another psychological construct relevant to coping with chronic illness is self-efficacy, defined as an individual’s belief in their ability to effectively manage challenging situations and health-related demands [25]. In the context of transplantation, self-efficacy may facilitate adherence to medical recommendations, promote adaptive coping with uncertainty, and support long-term psychological adjustment. At the same time, transplant recipients may experience a wide range of psychological symptoms—including somatization, obsessive–compulsive tendencies, depression, anxiety, interpersonal sensitivity, hostility, and paranoid ideation—which can complicate the adaptation process and negatively influence health behaviors and well-being [26,27].

### 1.3. Study Rationale and Study Aim

From a nursing perspective, psychological adaptation is particularly important because transplant nurses maintain continuous contact with recipients throughout the pre- and post-transplant trajectory. Nurses are often the first healthcare professionals to recognize early signs of psychological distress, difficulties with treatment adherence, or problems integrating the transplant experience into daily life. Understanding the psychological responses associated with transplantation can therefore inform nursing assessment, patient education, and psychosocial support strategies to improve long-term well-being and health outcomes among transplant recipients [4,28,29].

Despite growing evidence, most research is single-center and analyzes heart or kidney transplant recipients separately. They rarely combine transplant-specific psychological adaptation measured with the TxEQ and health-related quality of life. Also, no multicentre studies have been conducted in Central Europe. Addressing these gaps may improve understanding of the psychological mechanisms underlying successful post-transplant adaptation and facilitate the development of targeted psychosocial interventions.

Therefore, the present study aimed to examine associations between sociodemographic, psychological, and health-related quality-of-life variables and transplant-specific emotional responses.

## 2. Materials and Methods

### 2.1. Design

This multicentre cross-sectional observational study followed the Strengthening the Reporting of Observational Studies in Epidemiology (STROBE) guidelines. We consecutively recruited participants during routine outpatient follow-up visits at three transplant centers in Poland between September 2018 and December 2019. We invited all eligible recipients attending scheduled outpatient follow-up visits during the study period to participate. The investigation consisted of a written questionnaire that patients completed at the outpatient clinic during routine examinations. Patients were enrolled at age ≥18, after first single-organ transplantation, deceased donor transplantation, > one year after transplant, and with good cognitive and medical conditions. Exclusion criteria included age <18, more than one transplant, and inability to complete the questionnaire. The attending transplant physician or nurse assessed cognitive ability to complete the questionnaire during the outpatient visit as part of routine clinical assessment. Patients presenting with severe cognitive impairment, active psychiatric decompensation, or acute medical instability were not invited to participate.

This study was a part of the multicentre research project titled “Nonadherence with therapeutic recommendations among organ recipients—the occurrence of the phenomenon and its causes”.

### 2.2. Participants and Setting

**Figure 1:**
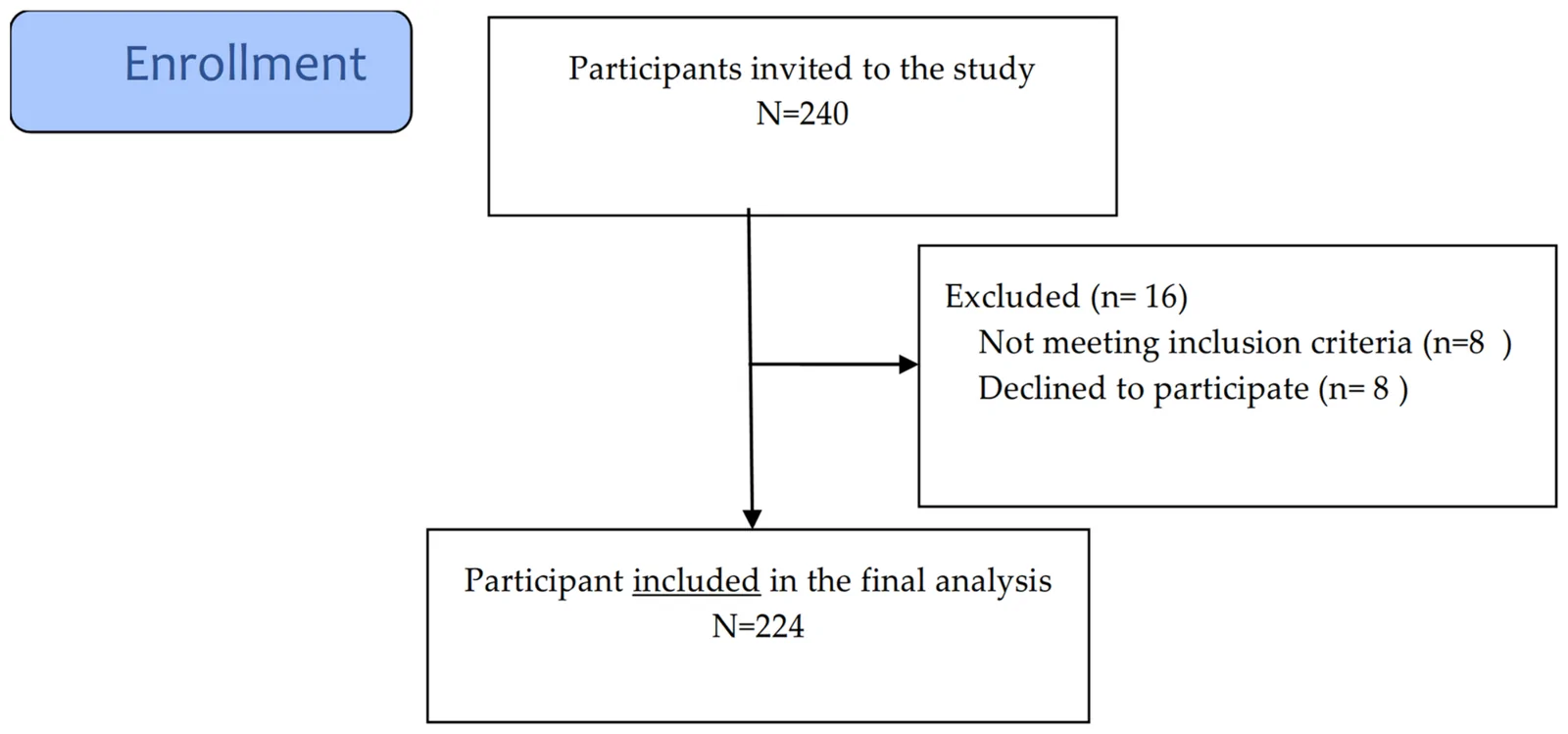
Flowchart of the patient selection.

The study was conducted in three transplant centers in Poland from 1 September 2018 to 31 December 2019. The study involved 240 patients after heart, kidney, liver, and lung transplantation. We invited all 240 eligible recipients to participate. Eight declined participation, and we excluded eight (three liver and five lung recipients) from the present analysis because subgroup sizes were insufficient. The final study population comprised 224 participants. After recruitment, only three liver and five lung recipients met the inclusion criteria. Because these sample sizes were insufficient for meaningful statistical analysis, we excluded these groups from the present secondary analysis. The final study group consisted of 224 participants. Figure 1 presents the participant selection process.

### 2.3. Instruments

The Transplant Effects Questionnaire (TxEQ) is a 23-item scale measuring transplant recipients’ emotional reactions. The TxEQ consists of 5 subscales assessing the following: worry about transplant (6 items), guilt regarding the donor (5 items), disclosure of having undergone transplantation (3 items), adherence to medical treatment (5 items), and responsibility to the donor, family, or medical staff (4 items). Participants respond to each item on a 5-point Likert scale, ranging from “strongly disagree” to “strongly agree” (score from 1 to 5). Higher scores indicate more worry about transplant, more disclosure, more feelings of guilt, more responsibility, and better adherence to medical treatment. TxEQ norm values are not available [18,30]. Internal consistency in the present study, assessed using Cronbach’s alpha, was—for worry about transplant—0.710, guilt—0.663, disclosure—0.715, adherence to medical treatment—0.757 and responsibility—0.782.

The Life Orientation Test-Revised (LOT-R) assesses dispositional optimism, a stable belief or predisposition to expect positive future outcomes. The test comprises 10 items with a 5-point Likert scale from 1 (completely disagree) to 5 (completely agree). Three statements were phrased optimistically and three pessimistically. A higher LOT-R score indicates a higher degree of dispositional optimism. We converted raw scores into standardized sten scores using normative values published by Juczyński (2012) [31]. Scores of 1–4 sten indicated low dispositional optimism, 5–6 average optimism, and 7–10 high optimism. The Polish version of the test had a reliability of 0.716 (Cronbach’s alpha).

General Self-Efficacy Scale (GSES). This self-report scale measures self-efficacy and consists of 10 items. Scoring of each item is from 1 (not at all true) to 4 (exactly true). The total score is calculated by summing all items. For the GSES, the total score ranges from 10 to 40, with higher scores indicating greater self-efficacy [32]. Internal consistency (Cronbach’s α) was 0.879.

Hospital Anxiety and Depression Scale (HADS) by A. S. Zigmond and R. P. Snaith, adapted by M. Majkowicz and K. de Walden-Gałuszko, was used to assess the intensity of anxiety and severity of depression. The HADS scale consists of 2 independent subscales measuring the level of anxiety and severity of depression. Each subscale contains 7 statements about the subject’s current state, rated from 0 to 3 points. Interpretation of HADS scores (for anxiety and depression subscales separately): 0–7—no disorders, 8–10—borderline states, 11–21—disorders [33]. Internal consistency, assessed using Cronbach’s alpha, was 0.777 for anxiety and 0.811 for depression.

The Short Form 36 Health Questionnaire (SF-36) measured quality of life (QOL). It contains 36 items. The SF-36 measures 8 scales: physical functioning (10 questions, PF), bodily pain (2 questions, BP), role physical (4 questions RP), general health (5 questions, GH), vitality (4 questions, VT), social functioning (2 questions, SF), role emotional (3 questions, RE) and mental health (5 questions, MH). The SF-36 measures the Physical Component Summary (PCS) and Mental Component Summary (MCS). The final measurement included physical activity (Physical Component Summary [PCS]) and mental activity (Mental Component Summary [MCS]), obtained by summing the physical components (PF, RP, BP, VT) for PCS and the mental components (SF, RE, MH, GH) for MCS. PCS and MCS scores were calculated according to the standard scoring algorithm provided by the questionnaire developer. Scores ranged from 0–100. Lower scores indicate more disability, and higher scores indicate less disability [34]. Licenses were obtained to use the questionnaire (License number QM044403). Cronbach’s alpha coefficients for the Polish version revealed positive internal consistency (0.38–0.98).

We obtained permission to use the Polish validated versions where required.

### 2.4. Statistical Analysis

All analyses were performed using R version 4.4.2 (R Foundation for Statistical Computing, Vienna, Austria). We analyzed quantitative variables (i.e., numerical data) by calculating descriptive statistics, including mean, standard deviation, median, quartiles, minimum, and maximum values. We analyzed qualitative variables (i.e., non-numerical data) by calculating absolute and percentage frequencies for all categories. We assessed the distribution of quantitative variables using the Shapiro–Wilk test and visual inspection of histograms and Q-Q plots. The comparison of qualitative variables between groups was performed using the chi-square test or Fisher’s exact test when chi-square assumptions were not met.

Because most variables were not normally distributed, we compared quantitative variables among three or more groups using the Kruskal–Wallis test; if statistically significant differences were detected, we applied Dunn’s post hoc test. We used linear regression for univariate and multivariate analyses of potential predictors of quantitative variables. Results are presented as regression parameters with 95% confidence intervals.

Variables with *p* < 0.20 in the univariable analyses were entered into the multivariable models to avoid premature exclusion of potentially relevant covariates. Separate multivariable linear regression models were constructed for each TxEQ domain because all TxEQ subscale scores were analyzed as continuous dependent variables. We verified model assumptions, including linearity, normality of residuals, homoscedasticity, and multicollinearity, before interpretation. Multicollinearity was assessed using variance inflation factors (VIFs), and no evidence of multicollinearity was observed (all VIFs < 2.0). Missing data were handled using complete-case analysis. Overall, four participants (1.8%) were excluded from the multivariable analyses because of incomplete data. Given the low proportion of missing observations, substantial bias was considered unlikely. Statistical significance was set at *p* < 0.05.

### 2.5. Ethical Consideration

The Bioethics Committee approved the study (Approval No. KBKA/63/O/2018), and we conducted it in accordance with the Declaration of Helsinki. All participants provided informed consent.

## 3. Results

### 3.1. Demographic and Baseline Characteristics

The final analysis included 224 transplant recipients: 107 heart and 117 kidney transplant recipients. Complete-case analysis excluded 4 participants (1.8%) from the multivariable models because they had missing values in at least one explanatory variable. The mean age was 51.8 years (SD 14.5), and the median time since transplantation was 7 years. Heart transplant recipients were significantly older than kidney recipients and had a longer post-transplant follow-up. In addition, the proportion of women differed significantly between transplant groups, whereas place of residence did not. Table 1 presents detailed data.

### 3.2. Transplant-Specific Psychological Adaptation

Recipients demonstrated high adherence to treatment (M = 4.22, SD = 0.71), felt moderately responsible for the transplanted organ (M = 3.21, SD = 1.01), and were moderately worried about their transplanted organ (M = 3.05, SD = 0.77). They experienced little guilt regarding the donor (M = 2.18, SD = 0.72) and had no difficulty in disclosing their identity as a transplant recipient (M = 1.84, SD 0.74). Adherence had the highest mean score, whereas disclosure was the lowest, indicating generally good adherence and little reluctance to disclose transplant status.

Comparison with the standard sample of the SF-36 representing the general population [34] shows that transplant patients score worse in both components of the SF-36 scale—Mental Component Summary (46.95, SD 9.66) and Physical Component Summary (46.04, SD 8.40). Personal resources included dispositional optimism and self-efficacy. The mean level of dispositional optimism among patients was within the range of Polish norms and at an average level (14.94, SD 4.48), whereas self-efficacy was higher than norms (30.13, SD 6.36). The mean scores of anxiety and depression do not indicate disorders. However, 20.68% of the study group presented with symptoms of anxiety and 14.22% with symptoms of depression. We found three statistically significant differences: Guilt regarding the donor, dispositional optimism, and the Physical Component of the SF-36. These variables were higher in patients after kidney transplant. Table 2 presents all results.

### 3.3. Univariate Analyses

In univariable analyses, association patterns differed between heart and kidney transplant recipients. Among heart transplant recipients, anxiety and depressive symptoms were associated with several TxEQ domains, whereas among kidney transplant recipients these associations were observed primarily for the worry domain. Psychological resources, including optimism, self-efficacy and mental health-related quality of life, were associated with selected TxEQ domains in both recipient groups. Associations with demographic variables were limited and domain-specific. Table 3 provides detailed data.

### 3.4. Multivariable Analyses

#### 3.4.1. Heart Transplant Recipients

Among heart transplant recipients, multivariable linear regression identified only one independent factor associated with transplant-specific adaptation. Better mental health-related quality of life (SF-36 MCS) was independently associated with higher adherence to medical treatment (β = 0.025, *p* = 0.003). No independent associations were observed for the worry, guilt, disclosure, or responsibility domains (*p* > 0.05).

#### 3.4.2. Kidney Transplant Recipients

Among kidney transplant recipients, transplantation at the UCK center was independently associated with higher donor-related guilt (β = 0.483, *p* < 0.05). Higher disclosure scores were independently associated with male sex (β = 0.280), transplantation at the UCK center (β = 0.374), and shorter time since transplantation (β = −0.011 per year; all *p* < 0.05). We found no significant independent associations for worry, responsibility, or adherence domains Table 4.

## 4. Discussion

This study evaluated factors associated with transplant-specific psychological adaptation among heart and kidney transplant recipients.

### 4.1. Principal Findings

This multicenter study showed that overall psychological adaptation among heart and kidney transplant recipients was generally satisfactory. Adherence represented the strongest domain of transplant-specific adaptation, whereas donor-related guilt and disclosure difficulties were relatively infrequent. However, symptoms of anxiety and depression remained significantly associated with less favorable transplant-related emotional responses. These findings suggest that heart and kidney transplant recipients showed broadly similar patterns of transplant-specific psychological adaptation. Several psychological variables were associated with selected domains of adaptation; however, owing to the cross-sectional design, these findings should be interpreted as associations rather than evidence of causal relationships.

### 4.2. Psychological Adaptation After Heart and Kidney Transplantation

Our results showed that recipients demonstrated high adherence to treatment, felt moderately responsible for the transplanted organ, and were moderately worried about it. They experienced little guilt regarding the donor and had no difficulty in disclosing their identity as a transplant recipient. Adherence had the highest mean score, whereas disclosure was the lowest, indicating generally good adherence and little reluctance to disclose transplant status.

Comparing organ groups helps identify differences in how people process the transplantation experience and adjust. In our study group, we did not observe differences between transplanted organ and psychological adaptation, except for guilt regarding the donor. One possible explanation for the similarity observed between heart and kidney transplant recipients is that long-term psychosocial adaptation appears to be associated with common challenges. Lifelong immunosuppressive therapy, continuous monitoring, uncertainty regarding graft function, and the need to integrate transplantation into everyday life create comparable psychological demands irrespective of organ type. As recipients move beyond the early postoperative period, disease-specific concerns may gradually diminish, whereas long-term coping resources, emotional regulation, and self-management become increasingly important determinants of psychological adaptation. These findings support the growing view that successful transplantation is a chronic adaptation process rather than a single therapeutic event. Although transplantation restores organ function, recipients continue to negotiate uncertainty, altered health identity, lifelong pharmacotherapy, and continuous interaction with healthcare services. Consequently, long-term adaptation increasingly depends on psychological flexibility and self-management rather than on organ-specific clinical characteristics [4,6,29]. Our findings are consistent with previous studies using the TxEQ, which consistently demonstrated high adherence, moderate levels of transplant-related worry and responsibility, and relatively low donor-related guilt among long-term transplant recipients. Similar patterns have been observed in German, Swedish, and Polish cohorts despite differences in healthcare systems and transplant populations [24,35,36].

Our findings suggest that selected psychological resources may be associated with transplant-specific psychological adaptation in clinically stable recipients, although prospective longitudinal studies are needed to confirm these associations. Contemporary psychological models increasingly conceptualize post-transplant adaptation as a resilience-driven process rather than merely the absence of emotional distress. Resilience facilitates acceptance of chronic illness, promotes adaptive coping, and enables recipients to maintain psychological stability despite persistent medical uncertainty. Recent reviews suggest that resilience interacts closely with optimism, self-efficacy, and perceived social support, thereby influencing treatment adherence and long-term psychosocial adjustment [6,25,29]. In our study, personal resources included dispositional optimism and self-efficacy. The observed levels of optimism and self-efficacy suggest that psychological resources may buffer the emotional consequences of lifelong transplantation. Previous studies consistently demonstrate that fear of graft loss is one of the dominant emotional experiences after transplantation [37,38,39]. Our results are consistent with these observations. Although mean HADS scores did not indicate clinically significant disorders, one in five recipients presented clinically relevant anxiety symptoms, and approximately one in seven reported depressive symptoms. These findings suggest that subclinical emotional distress remains common even among clinically stable recipients. A growing body of evidence indicates that emotional and psychological states directly affect treatment adherence, stress management, and, indirectly, clinical outcomes. Conversely, depression and anxiety may hinder the adaptation process [4,40]. Assessing resources and negative emotions is essential for designing psychological interventions [27]. Anxiety likely reflects persistent uncertainty about graft survival rather than current clinical instability. Even clinically stable recipients frequently report fear of rejection, fear of graft loss, and concerns regarding future health. Such uncertainty is a major psychological burden after transplantation and may persist for many years despite satisfactory medical outcomes [4,15,39].

### 4.3. Psychological Factors Associated with Transplant-Specific Adaptation

Psychological functioning after an organ transplant involves balancing emotional distress, medication adherence, and life uncertainty. Key factors influencing adjustment include pre-existing mental health, social support systems, and coping mechanisms [4].

In multivariable analyses, relatively few independent associations were found. Among heart transplant recipients, better mental health-related quality of life was independently associated with greater adherence. In kidney transplant recipients, transplant center was associated with higher donor-related guilt; disclosure was associated with male sex, transplantation center, and shorter time since transplantation.

Mental health-related quality of life emerged as the only independent association with adherence among heart transplant recipients. This finding suggests that recipients experiencing better emotional well-being may possess greater motivation and cognitive resources to maintain complex immunosuppressive regimens. Conversely, impaired mental health may reduce treatment engagement through decreased motivation, cognitive overload, or maladaptive coping. Similar associations between mental well-being and adherence have been consistently reported in transplant populations and support the integration of psychological assessment into routine long-term follow-up. One possible explanation is that better mental health may facilitate engagement with complex treatment regimens; however, the cross-sectional design precludes conclusions regarding directionality or causality. Patients reporting better psychological well-being may therefore allocate greater cognitive resources to medication management, follow-up attendance, and self-care behaviors required after transplantation.

The observed center-level differences may reflect variation in patient education, communication style, or psychosocial support; however, these variables were not measured, and therefore these explanations remain speculative. Survivor’s guilt can morph into post-traumatic stress disorder [41]. Donor-related guilt remains one of the most unique psychological reactions following transplantation. Although average guilt levels were relatively low in the present study, the association with transplant center suggests that contextual factors, including educational practices, communication styles, or cultural attitudes toward organ donation, may influence recipients’ emotional processing. Future multicenter studies should investigate whether standardized psychosocial education reduces variability in donor-related guilt across transplant programs. Donor-related guilt has increasingly been recognized as an existential rather than psychopathological phenomenon. Feelings of indebtedness toward the donor may coexist with gratitude and may even promote responsible health behaviors. Nevertheless, excessive guilt may also impair psychological well-being, highlighting the need for individualized psychological counseling [12,20,35,36].

Giri et al. studied the impact of TxEQ on various parameters affecting the health of kidney transplant recipients. They found that the mean score of worry about transplantation was significantly higher among older age groups, as well as among recipients with >24 months since transplant. Males reported significantly higher mean guilt toward donors. Mean adherence scores were significantly higher among older age groups and those with higher education levels [42]. Perceived responsibility scores were higher in the older age group [40]. In our study, univariable analyses showed that responsibility was positively associated with time since transplantation, whereas donor-related guilt showed a weak positive association with transplant duration among heart recipients.

In Scheel et al., all TxEQ subscales except “adherence” were associated with mental health-related quality of life but not with physical health-related quality of life [36].

Worry about transplantation can also be associated with fear of transplant rejection [38,39].

Differences between transplant centers probably reflect variations in patient education, communication style, psychosocial support availability, and organizational culture rather than biological differences among recipients. Although speculative, this observation suggests that transplant programs themselves may influence long-term psychological adaptation and deserve further investigation [42,43]. The relatively small number of independent predictors identified in multivariable models may reflect the multidimensional nature of psychological adaptation. Numerous biological, psychological, and social factors likely influence individual emotional responses after transplantation, which cross-sectional analyses cannot fully capture. Consequently, psychological adaptation should be viewed as a dynamic construct rather than a consequence of isolated demographic or clinical variables.

These observations are also consistent with contemporary adaptation theories, which describe transplantation as a process of reconstructing personal identity and integrating chronic illness into everyday life. Successful adaptation requires recipients to redefine their perception of health, autonomy, and future expectations while maintaining confidence in long-term graft survival [8,27,29]. Our findings further support the increasing role of patient-reported outcome measures in transplant follow-up. Instruments such as the TxEQ complement traditional clinical indicators by identifying recipients who are medically stable but continue to experience substantial psychological burden that may otherwise remain unrecognized [1,3,43].

### 4.4. Clinical Implications for Nursing Practice

The present findings support the potential value of routine psychosocial assessment during long-term transplant follow-up. Because this observational study did not evaluate psychosocial interventions, the effectiveness of structured nurse-led programs cannot be inferred from the present data. Future prospective and interventional studies should determine whether systematic psychosocial screening and nurse-led supportive interventions improve adherence, quality of life, and transplant-specific psychological adaptation. Our findings support routine psychosocial assessment performed by transplant nurses and provide a rationale for future intervention studies rather than suggesting proven effectiveness.

Routine psychological screening during outpatient follow-up may therefore allow nurses to identify recipients at risk of maladaptive adjustment. Nurses can also support psychological adaptation through patient education, emotional support, and referral to specialized psychological services when needed. Interventions that strengthen personal resources—such as self-efficacy, coping skills, and resilience—may improve recipients’ ability to manage the ongoing demands of transplantation, including lifelong immunosuppressive therapy and fear of graft rejection. Because transplant nurses have repeated contact with recipients throughout lifelong follow-up, they are ideally positioned to recognize subtle changes in emotional functioning before clinically significant psychological disorders develop. Our findings support future evaluation of structured nurse-led psychosocial support programs [29]. Incorporating routine transplant-specific psychological assessments such as TxEQ, HADS, and quality-of-life measures into nurse-led outpatient care may therefore facilitate earlier intervention and more individualized supportive care [28,29,44].

### 4.5. Strengths and Limitations

Several limitations should be acknowledged. First, the cross-sectional design precludes conclusions regarding temporal or causal relationships. Second, although the multicenter design increased the diversity of the study population, all participating centers were in Poland, which may limit the transferability of the findings to other healthcare systems. Third, participants were recruited during routine outpatient follow-up and represented clinically stable transplant recipients, introducing potential selection bias. Fourth, subgroup analyses resulted in moderate sample sizes, which may have reduced statistical power to detect weaker associations. Fifth, although multivariable models adjusted for selected covariates, residual confounding cannot be excluded. Finally, multiple statistical comparisons increase the possibility of type I error despite the prespecified modeling strategy. In addition, we did not include variables that could predict adaptation to life with a transplanted organ, such as PTSD [45] or resilience [46]. In our analysis, we also did not consider specific immunosuppression regimens, comorbid conditions, detailed social support structure, or pre-transplant psychiatric history.

## 5. Conclusions

In this multicenter cross-sectional study, several psychological variables were associated with selected domains of transplant-specific psychological adaptation among long-term heart and kidney transplant recipients. These findings support the potential value of incorporating psychosocial assessment into routine follow-up; however, prospective longitudinal and interventional studies are required to determine causal relationships and evaluate the effectiveness of structured nurse-led psychosocial interventions. Routine assessment of anxiety, mental health-related quality of life, and transplant-specific emotional responses may facilitate earlier identification of recipients requiring psychosocial support and enable more individualized nurse-led follow-up. Lifelong immunosuppression, uncertainty regarding graft survival, regular follow-up, and adaptation to chronic illness may outweigh disease-specific differences. Consequently, individual psychological resources appeared to show stronger associations with transplant-specific psychological adaptation than transplant type. Routine assessment of transplant-specific psychological adaptation using validated patient-reported outcome measures may facilitate earlier identification of recipients requiring psychosocial interventions and support individualized, nurse-led long-term transplant care. Prospective longitudinal studies should confirm these findings.

## Figures and Tables

**Table 1:** Characteristics of the study population.

Variable	Total (N = 224)	Kidney Transplant (n = 117)	Heart Transplant (n = 107)	*p*-Value
Age (years)	51.8 ± 14.5	49.8 ± 14.1	54.0 ± 14.6	0.008
Median (IQR)	54.5 (40–63)	49 (39–62)	60 (46.5–64)	
Male sex	154 (68.8)	68 (58.1)	86 (80.4)	<0.001
Urban residence	138 (61.6)	74 (63.2)	64 (59.8)	0.746
Rural residence	84 (37.5)	42 (35.9)	42 (39.3)	
*Missing data for residence (n = 2)*				
Time since transplantation (years)	7 (3–14)	4 (2.5–10.5)	11 (5–17)	<0.001

Values are presented as mean ± standard deviation (SD), median (interquartile range, IQR), or number (%). Continuous variables were compared using the Kruskal–Wallis test and categorical variables using the χ^2^ test or Fisher’s exact test, as appropriate.

**Table 2:** Statistical description of the study tools.

Parameter	All (N = 224)	Heart (N = 107)	Kidney (N = 117)	*p*
TxEQ worry	3.05 (±0.77)	2.93 (±0.77)	3.18 (±0.75)	0.073
TxEQ guilt	2.18 (±0.72)	2.08 (±0.81)	2.31 (±0.62)	0.017
TxEQ disclosure	1.84 (±0.74)	1.88 (±0.72)	1.80 (±0.78)	0.241
TxEQ adherence	4.22 (±0.71)	4.12 (±0.73)	4.28 (±0.71)	0.241
TxEQ responsibility	3.21 (±1.01)	3.17 (±0.97)	3.25 (±1.04)	0.274
HADS-depression	3.48 (±3.60)	3.66 (±3.86)	3.44 (±3.80)	0.307
HADS- depression cut-off ≥ 8 32 (14.2%)				
HADS-anxiety	4.68 (±3.58)	4.77 (±3.59)	4.61 (±3.53)	0.822
HADS-anxiety Cut-off ≥ 8 46 (20.53%)				
GSES	30.13 (±6.36)	30.46 (±4.54)	30.00 (±6.39)	0.580
LOT-R	14.94 (±4.48)	14.39 (±3.40)	15.53 (±3.66)	0.014
SF 36 MCS	46.95 (±9.66)	45.75 (±10.82)	47.95 (±8.45)	0.379
SF 36 PCS	46.04 (±8.40)	44.18 (±8.49)	47.60 (±8.21)	0.012

Values are presented as mean ± SD. Group comparisons were performed using the Kruskal–Wallis test. Abbreviations: GSES—General Self-Efficacy Scale, HADS—Hospital Anxiety and Depression Scale, LOT-R—Revised Life Orientation Test, MCS—Mental Component Scale, PCS, Physical Component Scale, SF36—Short Form-36, TxEQ, Transplant Effect Questionnaire.

**Table 3:** Univariable linear regression analyses of factors associated with transplant-specific psychological adaptation among heart and kidney transplant recipients.

Recipient Group	TxEQ Domain	Variable	β	95% CI	*p*
Heart	Worry	Self-efficacy (GSES)	−0.047	−0.080 to −0.014	0.006
		Anxiety (HADS-A)	0.072	0.035 to 0.108	<0.001
		Depression (HADS-D)	0.063	0.028 to 0.097	0.001
		SF-36 MCS	−0.021	−0.035 to −0.008	0.002
	Guilt	Time since transplantation	0.012	0.002 to 0.023	0.022
		Optimism (LOT-R)	−0.049	−0.096 to −0.002	0.039
		Anxiety	0.076	0.038 to 0.114	<0.001
		Depression	0.073	0.037 to 0.109	<0.001
		SF-36 PCS	−0.024	−0.042 to −0.006	0.009
		SF-36 MCS	−0.018	−0.032 to −0.004	0.013
	Disclosure	Male sex	0.369	0.029 to 0.709	0.034
		Optimism (LOT-R)	−0.057	−0.099 to −0.015	0.009
		Self-efficacy	−0.040	−0.073 to −0.008	0.015
		Anxiety	0.056	0.021 to 0.091	0.002
		Depression	0.074	0.037 to 0.111	<0.001
		SF-36 MCS	−0.015	−0.028 to −0.002	0.023
	Responsibility	Anxiety	0.061	0.013 to 0.109	0.013
		Depression	0.048	0.002 to 0.094	0.041
		SF-36 MCS	−0.021	−0.037 to −0.004	0.014
	Adherence	Optimism	0.031	0.004 to 0.058	0.022
		Self-efficacy	0.022	0.005 to 0.039	0.013
		SF-36 MCS	0.025	0.013 to 0.037	<0.001
Kidney	Worry	Time since transplantation	0.023	0.001 to 0.045	0.040
		Anxiety	0.049	0.010 to 0.089	0.015
		Depression	0.047	0.005 to 0.089	0.030
	Guilt	Transplant centre (UCK vs. KRK)	0.378	0.158 to 0.597	0.001
	Disclosure	Male sex	0.249	0.040 to 0.459	0.020
		Time since transplantation	−0.016	−0.030 to −0.002	0.029
		Transplant centre (UCK vs. KRK)	0.436	0.225 to 0.647	<0.001
	Responsibility	Time since transplantation	0.017	0.004 to 0.030	0.014
	Adherence	Optimism	0.030	0.005 to 0.055	0.020
		Self-efficacy	0.021	0.005 to 0.037	0.013

Values are univariable linear regression coefficients (β) with 95% confidence intervals. Variables meeting the predefined inclusion criterion (*p* < 0.20) for multivariable modelling are shown. Abbreviations: GSES—General Self-Efficacy Scale; HADS-A—Hospital Anxiety and Depression Scale, Anxiety; HADS-D—Hospital Anxiety and Depression Scale, Depression; LOT-R—Life Orientation Test-Revised; PCS—Physical Component Summary; MCS—Mental Component Summary; KRK—Kraków Transplant Center; UCK—University Clinical Center, Gdańsk.

**Table 4:** Multivariable linear regression models for transplant-specific psychological adaptation.

Recipient Group	TxEQ Domain	Independent Variable	β	95% CI	*p*
Heart	Adherence	SF-36 Mental Component Summary	0.025	0.009 to 0.041	0.003
Kidney	Guilt	Transplant center (UCK vs. KRK)	0.483	0.214 to 0.752	0.001
	Disclosure	Time since transplantation	−0.011	−0.020 to −0.002	0.018
		Male sex	0.280	0.070 to 0.490	0.010
		Transplant center (UCK vs. KRK)	0.374	0.111 to 0.637	0.006

Only variables remaining statistically significant in the final multivariable models are presented. Variables with *p* < 0.20 in univariable analyses were entered into multivariable models using a prespecified selection strategy. Regression assumptions (linearity, homoscedasticity, normality of residuals and multicollinearity) were verified before model interpretation. Maximum variance inflation factor (VIF) was below the accepted threshold, indicating no relevant multicollinearity. Abbreviations: β—unstandardized regression coefficient; CI—confidence interval; KRK—Kraków Transplant Centre; UCK—University Clinical Centre, Gdańsk.

## Data Availability

The data presented in this study are available on request from the author due to privacy restrictions.

## Abbreviations

The following abbreviations are used in this manuscript:

HADS	Hospital Anxiety and Depression Scale
GSES	General Self-Efficacy Scale
LOT-R	The Life Orientation Test-Revised
MCS	Mental Component Summary
PCS	Physical Component Summary
PROMs	Patient-reported outcome measures
PTSD	Post-traumatic stress disorders
QOL	Quality of life
TxEQ	Transplant Effects Questionnaire
SF-36	The Short Form 36 Health Questionnaire
